# A Potential Association between Ribonuclease 1 Dynamics in the Blood and the Outcome in COVID-19 Patients

**DOI:** 10.3390/ijms241512428

**Published:** 2023-08-04

**Authors:** Elisabeth Zechendorf, Christian Beckers, Nadine Frank, Sandra Kraemer, Carolina Neu, Thomas Breuer, Michael Dreher, Edgar Dahl, Gernot Marx, Lukas Martin, Tim-Philipp Simon

**Affiliations:** 1 Department of Intensive Care and Intermediate Care, University Hospital RWTH Aachen, 52074 Aachen, Germanytsimon@ukaachen.de (T.-P.S.); 2 Department of Pneumology and Intensive Care Medicine, University Hospital RWTH Aachen, 52074 Aachen, Germany; 3 RWTH Centralized Biomaterial Bank (RWTH cBMB) at the Institute of Pathology, University Hospital RWTH Aachen, 52074 Aachen, Germany

**Keywords:** COVID-19, SARS-CoV-2, RNase 1, kidney injury, biomarker

## Abstract

The COVID-19 pandemic caused by the new SARS-CoV-2 coronavirus is the most recent and well-known outbreak of a coronavirus. RNase 1 is a small endogenous antimicrobial polypeptide that possesses antiviral activity against viral diseases. In this study, we investigated a potential association between ribonuclease 1 and the outcome in COVID-19 patients and the impact of increased and decreased RNase 1 levels serum during the course of the disease. Therefore, two patient populations, Cohort A (*n* = 35) and B (*n* = 80), were subclassified into two groups, in which the RNase 1 concentration increased or decreased from time point one to time point two. We show that the RNase 1 serum levels significantly increased in the increasing group of both cohorts (*p* = 0.0171; *p* < 0.0001). We detect that patients in the increasing group who died had significantly higher RNase 1 serum levels at both time points in Cohort A (*p* = 0.0170; *p* = 0.0393) and Cohort B (*p* = 0.0253; *p* = 0.0034) than patients who survived. Additionally, we measured a significant correlation of RNase 1 serum levels with serum creatinine as well as creatinine clearance in the increasing and decreasing group at both time points of Cohort A. Based on these results, there is now good evidence that RNase 1 may play a role in renal dysfunction associated with ICU COVID-19 patients and that increasing RNase 1 serum level may be a potential biomarker to predict outcome in COVID-19 patients.

## 1. Introduction

Coronaviruses are a family of seven known single-stranded RNA viruses that can cause a range of illnesses, from mild upper respiratory disease to more severe respiratory illnesses [1,2]. The most recent and well-known outbreak of a coronavirus is the COVID-19 pandemic caused by the new SARS-CoV-2 coronavirus. SARS-CoV-2 has caused a severe health crisis with millions of confirmed cases and hundreds of thousands of deaths worldwide, so the World Health Organization (WHO) declared it a Public Health Emergency of International Concern on 30 January 2020 [1]. Since the emergence of COVID-19, research on coronaviruses has been strongly promoted and grown, and scientists and clinicians are working to better understand the virus and to develop effective treatments. However, there is still much to learn about coronaviruses and their general potential to cause further outbreaks in the future. In addition, new strains of the virus have emerged in the meantime, underscoring the need for continued research on the virus and its variants.

Ribonucleases are small endogenous antimicrobial polypeptides that possess antiviral activity against viral diseases. Several studies have demonstrated that RNase 1 and 2 possess antiviral properties against human immunodeficiency virus (HIV)-1 [3,4]. In addition to its antiviral activity, RNase 1 has high ribonucleolytic activity and thus plays a potential role in regulating the immune response by recognizing and degrading single- and double-stranded RNA molecules [5]. As a result of an inflammatory response and associated cell death, extracellular RNA (eRNA) is released. The eRNA binds to Toll-like receptors (TLRs) 3 and 7 and induces various signaling pathways involved in the initiation of the innate immune response [6,7,8]. Due to its ribonucleolytic activity, RNase 1 is thought to have the ability to recognize and degrade viral RNA, thus preventing its replication in host cells [9]. This could contribute to preventing an excessive or dysregulated immune response that can lead to severe disease progression. Ireland and colleagues showed in a study that RNase L has a protective role on a murine coronavirus mouse hepatitis beta CoV strain (MHV-JHM) [10].

COVID-19 has been shown to be associated with renal dysfunction [11,12,13]. In a previous study, we showed that patients with renal dysfunction had significantly higher RNase 1 levels after thoracoabdominal aortic aneurysm (TAAA) repair than patients without renal dysfunction [14]. However, the role of RNase 1 and its association with renal function in patients infected by SARS-CoV-2 is unknown.

In this study, we therefore measured the RNase 1 dynamics in the blood of patients with SARS-CoV-2 infection in two different cohorts. To determine the prognostic value of RNase 1 as a potential new biomarker in diseases induced by SARS-CoV-2 and its potential as a possible therapeutic target, we also investigated whether RNase 1 levels decrease or increase during disease progression. Furthermore, we correlate RNase 1 serum levels with 28-day mortality and other clinical parameters such as C-reactive protein (CRP), interleukin-6 (IL-6), and procalcitonin (PCT) of patients with increasing or decreasing RNase 1 serum levels. To investigate the role of RNase in SARS-CoV-2-induced renal dysfunction, we also investigated the correlation of RNase 1 levels with serum creatinine and creatinine clearance of patients with SARS-CoV-2 and the impact of increased and decreased RNase 1 levels during the course of the disease.

## 2. Results

### 2.1. Patient Characteristics

In the first cohort, 35 patients with SARS-CoV-2 infection were included (Table 1). Patients in whom the RNase 1 serum levels increased or decreased from day 2 to day 4 were categorized in one of two groups, the increasing (*n* = 22) and decreasing (*n* = 13) RNase 1 level groups. On average, the patients were 61 years old, and 74.29% were male. Only eight patients were diagnosed with diabetes mellitus, and all eight patients belonged to the increasing RNase 1 group (*p* = 0.0124). While the average time in ICU was 19 days, patients belonging in the increasing RNase 1 group were in the ICU for a mean of 23.5 days and those in the decreasing group were in the ICU only for a mean of 17 days (*p* = 0.0443). Further details of the patient characteristics of Cohort A can be found in Table 1.

In the second cohort (Cohort B), 80 patients with SARS-CoV-2 infection were included (Table 2). The patients of this group were also categorized in two groups, where the RNase 1 serum levels increased (*n* = 48) or decreased (*n* = 32) from day 1 to week 1. On average, the patients were 64 years old, and 63.75% were male. Forty-four patients were in the ICU, 31 patients belonged to the increasing RNase 1 group, and only 13 of these patients belonged to the decreasing group (*p* = 0.0351). The ICU patients of the increasing group were in the ICU for an average of 18 days, whereas the patients of the decreasing RNase 1 group were in the ICU for a mean of 21 days. Further details of the patient characteristics of Cohort B can be found in Table 2.

### 2.2. RNase 1 Serum Levels

**Figure 1:**
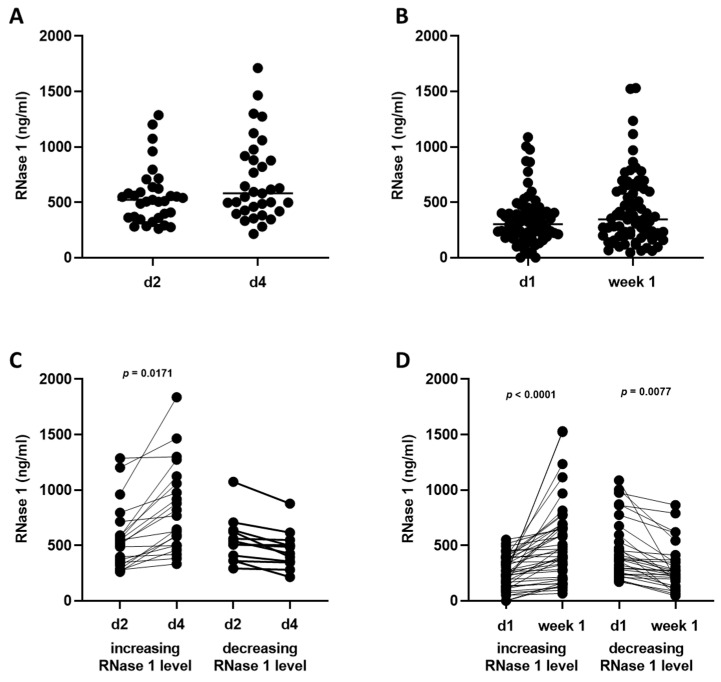
RNase 1 serum levels in Cohorts A and B. (**A**) RNase 1 serum levels of all patients in Cohort A on day 2 (d2) and day 4 (d4) and (**B**) Cohort B on day 1 (d1) and week 1 are presented. (**C**,**D**) The patients in the cohorts were grouped into two groups. In the first group, patients were included in which the RNase 1 serum levels increased (increasing RNase 1 levels), and in the second group, patients with decreasing RNase 1 serum levels (decreasing RNase 1 levels) were included. An unpaired *t*-test (two-tailed) was used for statistical analysis.

First, we wanted to analyze whether the RNase 1 serum levels increased or decreased in COVID-19 patients over two days (Figure 1A) or a week (Figure 1B). We could not detect a significant increase in RNase 1 serum levels in the different cohorts (Figure 1A,B). Therefore, we grouped the patients into two groups based on their increasing or decreasing RNase 1 serum levels. In Cohort A, 22 patients were detected in whom RNase serum levels increased (increasing group) from day 2 (564.4 ± 289.9 ng/mL) to day 4 (871.9 ± 431.7 ng/mL), whereas 13 patients in Cohort A had a decrease in RNase 1 serum levels (decreasing group) from day 2 (549.0 ± 207.7 ng/mL) to day 4 (462.4 ± 173.7 ng/mL). In Cohort B, an increase in RNase 1 serum levels (increasing group) from day 1 (260.3 ± 141.3 ng/mL) to week 1 (517.9 ± 352.9 ng/mL) was detected in 48 patients. In 32 patients of Cohort B, a decrease in RNase 1 serum levels (decreasing group) from day 1 (465.6 ± 268.2 ng/mL) to week 1 (294.9 ± 206.5 ng/mL) was measured. We showed that the RNase 1 serum levels significantly increased in the increasing group from day 2 to day 4 (*p* = 0.0171; Figure 1C). In Cohort B, we detected a significant increase in RNase 1 serum levels from day 1 to week 1, as well as a significant decrease in the decreasing group (*p* < 0.0001 and *p* = 0.0077; Figure 1D).

### 2.3. Correlation of RNase 1 Serum Levels with 28-Day Mortality

**Figure 2:**
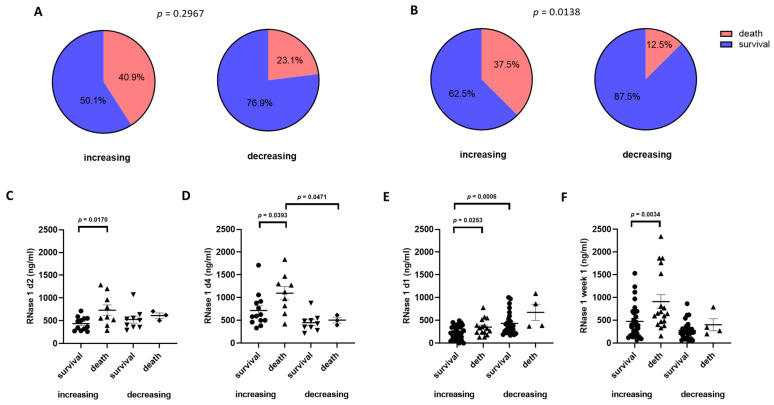
Association between RNase 1 serum levels and 28-day mortality. (**A**) Survival rate of all patients in Cohort A and (**B**) Cohort B grouped by increasing and decreasing RNase 1 serum levels in percentage. The correlation of RNase 1 serum levels with the mortality rate in COVID-19 patients in Cohort A on (**C**) day 2 and (**D**) day 4 as well as (**E**) in Cohort B on day 1 and (**F**) week 1 grouped in increasing and decreasing RNase 1 levels. Unpaired *t*-test (two-tailed) or one-way ANOVA was used for statistical analysis.

Next, we investigated the correlation between RNase 1 serum levels and 28-day mortality in COVID-19 patients. We show that in Cohort A, 40.9% of the patients died within 28 days in the increasing RNase 1 group, and only 23.1% of the patients were included in the deceasing group (Figure 2A). In Cohort B, we found that there were significantly more patients who died in the increasing group than in the decreasing group (*p* = 0.0138; Figure 2B). Furthermore, we analyzed the RNase 1 serum levels of the different groups and showed that patients in Cohort A of the increasing group who died had significantly higher RNase 1 serum levels on day 2 than patients who survived (*p* = 0.0170; Figure 2C). On day 4, we measured significantly higher RNase 1 serum levels in patients who died later in the increasing group compared with surviving patients in the increasing group (*p* = 0.0393) and dying patients in the decreasing group (*p* = 0.0471; Figure 2D). In the decreasing group, no significant difference between the RNase 1 concentrations in surviving or dying patients was detected in Cohort A on day 2 and 4 (Figure 2C,D). In Cohort B, we also measured significantly higher RNase 1 serum levels in dying patients of the increasing group compared with surviving patients of the increasing group on day 1 and week 1 (*p* = 0.0253 and *p* = 0.0034; Figure 2E,F). In contrast to Cohort A, we detected significantly higher RNase 1 concentrations in the serum of surviving patients in the decreasing group compared to surviving patients in the increasing group on both days in Cohort B (*p* = 0.0006; Figure 2E).

### 2.4. RNase 1 Serum Levels Correlate with Creatinine Serum Levels and Creatinine Clearance in COVID-19 Patients

**Figure 3:**
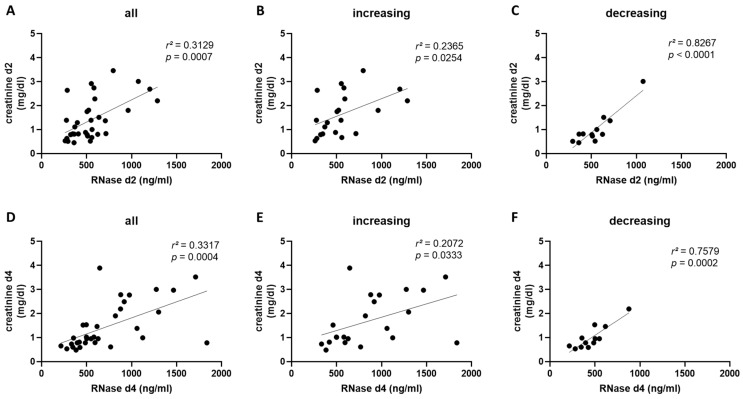
Correlation of RNase 1 and creatinine serum levels in Cohort A. (**A**) Correlation of RNase 1 and creatinine serum levels of all COVID-19 patients in Cohort A on day two is presented. The correlation of RNase 1 and creatinine serum levels two days after COVID-19 diagnosis in patients with (**B**) increasing or (**C**) decreasing RNase 1 serum levels from day 2 to day 4 is shown. (**D**) The correlation of RNase 1 and creatinine serum levels of all COVID-19 patients in Cohort A four days after COVID-19 infection is presented. The correlation of RNase 1 and creatinine serum levels four days after COVID-19 diagnosis in patients with (**E**) increasing or (**F**) decreasing RNase 1 serum levels from day 2 to day 4 is shown. Simple linear regression was used for statistical analysis.

To investigate the role of RNase 1 in kidney function/injury in patients with SARS-CoV-2 infection, we explored correlation of serum creatinine levels with RNase 1 serum levels in the different groups and time points. We measured a significant correlation between the creatinine and RNase 1 serum levels in all patients on day two (*p* = 0.0007) and day four (*p* = 0.0004) after COVID-19 diagnosis (Figure 3A,D). Next, we grouped the patients according to increasing and decreasing RNase 1 serum levels and correlated the creatinine serum levels again with the RNase 1 levels. We also detected a significant correlation on day two and day four in the increasing group (*p* = 0.0254 and *p* = 0.0333; Figure 3B,E). Interestingly, in the decreasing group, we detected a higher significance in the correlation between creatinine and RNase 1 serum levels on both days (*p* < 0.0001 and *p* = 0.0002; Figure 3C,F).

**Figure 4:**
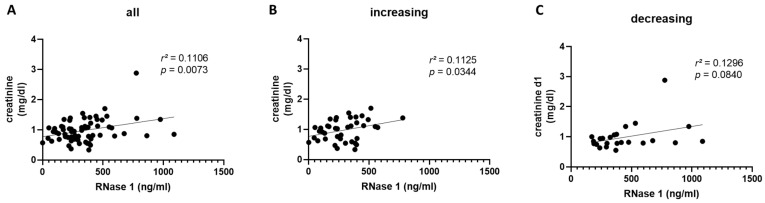
The correlation of RNase 1 and creatinine serum levels in Cohort B. (**A**) The correlation of RNase 1 and creatinine serum levels of all COVID-19 patients in Cohort B on the day of COVID-19 infection is shown. The correlation of RNase 1 and creatinine serum levels in patients with (**B**) increasing or (**C**) decreasing RNase 1 serum levels from day 1 to week 1 is presented. Simple linear regression was used for statistical analysis.

We also investigated the correlation between serum creatinine levels and RNase 1 serum levels in the second cohort on day one. We measured a significant correlation between the creatinine and RNase 1 serum levels in all patients on day one (*p* = 0.0073; Figure 4A). After grouping the patients of the second cohort into an increasing and decreasing group, we detected, in contrast to the first cohort, only a significant correlation between the creatinine and RNase 1 serum levels in the increasing group (*p* = 0.0344; Figure 4B).

**Figure 5:**
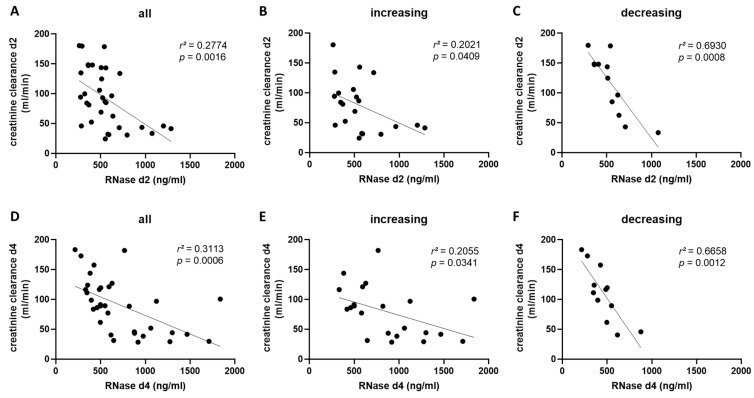
Correlation of RNase 1 serum levels and creatinine clearance on day 2 and 4 in Cohort A. (**A**) The correlation of RNase 1 and creatinine clearance of all COVID-19 patients in Cohort A on day two is presented. The correlation of RNase 1 and creatinine clearance two days after COVID-19 diagnosis in patients with (**B**) increasing or (**C**) decreasing RNase 1 serum levels from day 2 to day 4 is shown. (**D**) The correlation of RNase 1 and creatinine clearance of all COVID-19 patients in Cohort A four days after COVID-19 infection is presented. The correlation of RNase 1 and creatinine clearance four days after COVID-19 diagnosis in patients with (**E**) increasing or (**F**) decreasing RNase 1 serum levels from day 2 to day 4 is shown. Simple linear regression was used for statistical analysis.

Furthermore, we explored the correlation of creatinine clearance with RNase 1 serum levels in the different groups and time points. In Cohort A, we measured a significant negative correlation between creatinine clearance and RNase 1 serum levels in all patients on day two (*p* = 0.0016) and day four (*p* = 0.0006) after COVID-19 diagnosis (Figure 5A,D). After grouping patients according to increasing and decreasing RNase 1 serum levels, we detected a higher significant correlation between creatinine clearance and RNase 1 serum levels on both days in the increasing group (*p* = 0.0008 and *p* = 0.0012; Figure 5C,F) than in the decreasing group (*p* = 0.0409 and *p* = 0.0341; Figure 5B,E).

**Figure 6:**
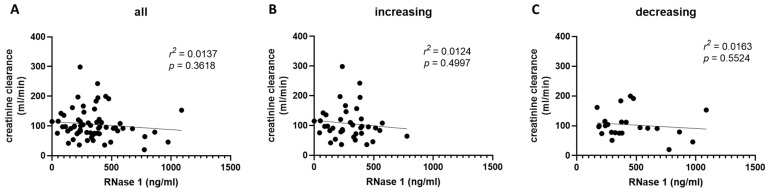
Correlation of RNase 1 and creatinine clearance on the day of diagnosis in Cohort B. (**A**) The correlation of RNase 1 and creatinine clearance of all COVID-19 patients in Cohort B on the day of COVID-19 infection is shown. The correlation of RNase 1 and creatinine clearance in patients with (**B**) increasing or (**C**) decreasing RNase 1 serum levels from day 1 to week 1 is presented. Simple linear regression was used for statistical analysis.

In Cohort B, we did not detect a correlation between creatinine clearance and serum RNase 1 levels, neither in all patients nor in different subgroups and time points (Figure 6).

### 2.5. Time Course of Biomarkers and Scores over 14 Days and the Correlations of RNase 1 Levels with Clinical Parameters of ICU Patients with SARS-CoV-2 Infection

**Figure 7:**
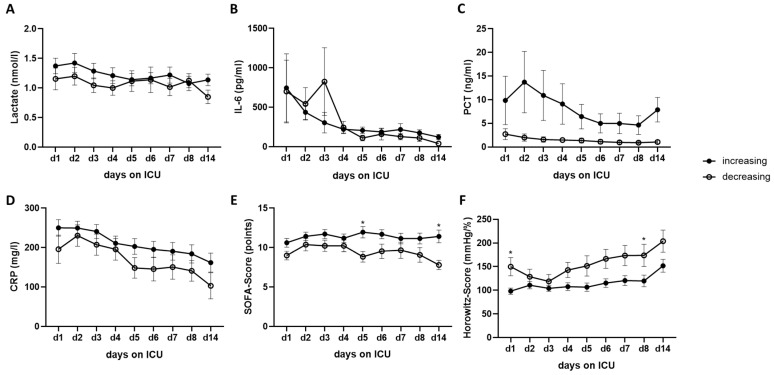
The time course of different biomarkers over 14 days in COVID-19 patients in Cohort A. Presented are (**A**) lactate, (**B**) IL-6, (**C**) PCT, (**D**) CRP, (**E**) the SOFA score, and (**F**) the Horowitz score over 14 days in COVID-19 patients grouped into increasing and decreasing RNase 1 serum levels. Ordinary two-way ANOVA was used for multiple comparisons (* *p* < 0.05).

ICU patients with SARS-CoV-2 infection (Cohort A), grouped into increasing and decreasing RNase 1 serum levels, were evaluated for various biomarkers and scores (lactate, IL-6, PCT, CRP, sepsis-related organ failure assessment (SOFA) score, and Horowitz score) over a 14-day period. No relevant differences could be detected between the groups (Figure 7).

To investigate the impact of increased and decreased RNase 1 levels serum during the course of the disease, we correlated RNase 1 serum level with various clinical parameters (Table 3). We detected, that RNase 1 level on day 2 of the increasing group correlate positive with PCT (*p* = 0.0132). Furthermore, we also measured a positive correlation with the days of dialysis and a negative correlation with diuresis (*p* = 0.0199; *p* = 0.0044) in the increasing group on day 2. On day 4, we detected a negative correlation with CRP and diuresis in the increasing group (*p* = 0.0200; *p* = 0.0013). A significant positive correlation with leucocytes and days of dialysis was also measured in the increasing group on day 4 (*p* = 0.0167; *p* = 0.0046). In the decreasing group, no significant correlations were detected (Table 3).

## 3. Discussion

Several studies have demonstrated that RNase 1 possesses antiviral properties against HIV-1 [3,4]. Therefore, it has been postulated that RNases may be candidate drugs for host defense and could provide an alternative means to combat viral infections [9,15]. In this study, we demonstrated for the first time that RNase 1 serum levels play a potential role in patients with SARS-CoV-2 infection.

We could not detect changes in RNase 1 serum levels over time in both cohorts when all patients were analyzed together. However, after grouping patients in the two cohorts into an increasing and a decreasing group, we found that RNase 1 serum levels significantly increased in Cohort A from day 2 to day 4 and in Cohort B from day 1 to week 1. In Cohort A, a decrease in RNase 1 levels was observed in the decreasing group. The decrease in RNase 1 serum levels was even significant in Cohort B in the decreasing group from day 1 to week 1. It is possible that RNase 1 levels in Cohort A would also decrease significantly from the first to the second time point if the second measurement had been performed at a later time point, as in Cohort B. Because the patients in Cohort A were only ICU patients, whereas Cohort B also included patients who were not in the ICU, the two patient cohorts were different in the time points.

We showed that patients in the increasing group of Cohort A stayed in the ICU significantly longer than patients in the decreasing group. Similarly, patients in the increasing group of the second cohort were in the ICU significantly more often than patients in the decreasing group. In both cohorts, a higher mortality rate was observed in patients of the increasing group compared with patients with decreasing RNase 1 levels from time point 1 to 2. These data are in line with a previous study showing that patients with significantly higher serum RNase 1 levels 12 h after TAAA repair had a higher mortality rate than patients with lower RNase 1 levels [14].

In previous studies, it was shown that serum RNase 1 serum levels are associated with the development of renal failure and positively correlated with serum creatinine in patients with leukemia [16]. Consistent with this study, we also measured a significant positive correlation of RNase 1 serum levels with serum creatinine in all groups of Cohort A in the present study. In addition, we measured a positive correlation with the days of dialysis and a negative correlation with diuresis (*p* = 0.0199; *p* = 0.0044) in the increasing group on day 2. On day 4, we also detected a significant positive correlation with days of dialysis and a negative correlation diuresis in the increasing group (*p* = 0.0046; *p* = 0.0013). This suggests that increasing RNase 1 levels in patients with SARS-CoV-2 infection are associated with renal injury.

Although more patients died in the increasing group and no significant correlations with other clinical parameters were observed, a stronger positive correlation between creatinine levels and RNase 1 levels was measured in the decreasing group at both day 2 and day 4. Consistently, we showed that higher RNase 1 levels were associated with lower creatinine clearance. Moreover, there was a stronger correlation between RNase 1 levels and creatinine clearance in the decreasing group than in the increasing group. Consistent with this, our research group showed in a previous study that patients with renal dysfunction had significantly higher RNase 1 levels after TAAA repair than patients without renal dysfunction [14]. In addition, patients with higher serum RNase 1 levels were more likely to develop stage 3 acute kidney injury 48 h after surgery [14]. Furthermore, sepsis patients with renal dysfunction were found to have significantly higher RNase 1 levels than patients without renal dysfunction [17]. Additionally, Martin et al. showed that patients with sepsis have significantly higher serum RNase 1 levels than healthy volunteers [17]. Indeed, at days 5 and 14, we measured significantly higher SOFA scores in the increasing group than in the decreasing group.

SARS-CoV-2 infection can cause ARDS, which is defined according to the 2012 Berlin ARDS diagnostic criteria [18,19,20]. The severity of ARDS depends on the Horowitz quotient (PaO2/FiO2) [20]. Interestingly, a significant difference in Horowitz score was observed at days 1 and 8, which may suggest that increasing RNase 1 levels over time in COVID-19 patients are associated with lung injury. However, this could not be confirmed using other clinical parameters and biomarkers, where no differences were found. It could only be shown that the values of the increasing group were higher compared with the decreasing group in all biomarkers. This suggests that increasing RNase 1 levels may be associated with worse outcomes in SARS-CoV-2 patients.

Several studies have described that males with SARS-CoV-2 infection have a worse outcome than female patients [21,22,23]. However, in this study, we did not detect any association between RNase 1 concentration and worse outcome in relation to biological sex.

## 4. Materials and Methods

### 4.1. Study Design/Population

In this study, two different patient populations were analyzed. All serum samples were collected, based on approval by the Ethics Committee of the University Hospital RWTH Aachen (EK 100/20, proofed on the 7 April 2020 and EK 080/20, proofed on the 27 March 2020). All patients or their legal representatives provided written informed consent. The serum samples of the first cohort (Cohort A) were collected between March and April 2020. Thirty-five patients with positive SARS-CoV-2 PCR results and intensive care admission were included in this study. This patient population has been described in previous studies by our research group [24,25,26]. The serum samples of patients with COVID-19 infection in the second study population were collected between April 2020 and May 2021. In this cohort, 80 patients were included (Cohort B). Patients who were younger than 18 years of age, pregnant, or under palliative care were excluded. Identification of infection was carried out using real-time reverse transcription PCR (RT-PCR). All parameters, including demographics, vital signs, laboratory values, blood gas analyses, and organ support, were extracted from the patient data management system (Intellispace Critical Care and Anesthesia (ICCA) system, Philips, Amsterdam, Netherlands).

The patient populations were subclassified into an “increasing group”, in which the RNase 1 concentration increased over the two time points, and a “decreasing group”, in which the RNase 1 concentration decreased from time point one to time point two, similar to those in Bleilevens et al. [24].

### 4.2. Serum Sampling

Serum samples of Cohort A were collected one and three days (d2 and d4) after SARS-CoV-2 infection was confirmed via PCR. In Cohort B, serum samples were collected on the day when the positive PCR result was available (d1) and one week (week 1) after SARS-CoV-2 infection was confirmed. After 10 min of coagulation, serum samples were centrifuged at 3000 rpm for 10 min at room temperature and stored at −80 °C until RNase 1 serum levels were measured.

### 4.3. Human Enzyme-Linked Immunosorbent Assay

Levels of RNase 1 in human serum were determined using a commercial ELISA kit (#SEK13468; Sino Biological Inc., Beijing, China) according to the manufacturer’s instructions. Briefly, a 96-well microplate was coated with capture antibody and incubated overnight at 4 °C. The antibody solution was discarded, and the microplate was washed with at least 300 µL of wash buffer three times. Next, the plate was blocked by adding 300 µL of blocking buffer to each well, incubated at room temperature for 1 h, and washed. Afterward, the standard and samples were added in duplicate. After 2 h of incubation at room temperature, the washing step was repeated, and 100 µL of a detection antibody was added to each well and incubated for 1 h at room temperature. The microplate was washed three times, and 200 µL of a substrate solution was added to each well and incubated for 20 min at room temperature; 50 µL of stop solution was added to stop the reaction. For analysis, the optical density was measured at 450 nm and 570 nm as a reference using a microplate reader (Tecan Group, Männedorf, Switzerland).

### 4.4. Statistics

Individual values are presented as scatter plots. Lines represent the mean with SEM. To compare patient characteristics of the decreasing and increasing RNase 1 level groups, as well as RNase 1 serum levels on day two/one and day four/week one, unpaired *t*-tests were used. To assess the association between 28-day mortality (death/survival) and RNase 1 serum levels in the increasing and decreasing groups at each time point, one-way ANOVA was used. For each point in time, a simple linear regression was applied to assess the association between the outcome variables serum creatinine level and creatinine clearance with RNase 1 serum levels.

## 5. Limitation/Conclusions

Our study is limited by the different time points at which RNase 1 levels were determined in the two cohorts. In Cohort A, the RNase 1 levels were measured on days 2 and 4, and in Cohort B, the RNase levels were measured on day 1 and week 1, making it difficult to compare the two cohorts. Further investigation in a larger cohort in which RNase 1 serum levels are measured at multiple time points over a week should be performed to confirm our data. In addition, our measurements were limited to serum RNase 1 levels; measurement of RNase activity and determination of serum eRNA concentration would strengthen the results reported here.

In conclusion, we showed that a higher mortality rate was observed in patients with SARS-CoV-2 infection in patients with increasing RNase 1 levels from time point one to two. Moreover, in this study, we found significant positive correlations of serum RNase 1 levels with several biomarkers associated with renal injury in the increasing group of Cohort A. Additionally, at days 5 and 14, we measured significantly higher SOFA scores in the increasing group. Based on these results, there is now good evidence that increasing RNase 1 serum levels may play a role in renal dysfunction associated with COVID-19 and that increasing RNase 1 serum levels may be a potential biomarker to predict outcome in ICU patients with SARS-Cov-2 infection. However, more studies are needed to identify the underlying mechanisms. 

## Figures and Tables

**Table 1:** Patient characteristics of Cohort A.

	All (*n* = 35)	Increasing RNase 1 Levels (*n* = 22)	Decreasing RNase 1 Levels (*n* = 13)	*p*-Value
Age (year) (IQR)	61.00 (56.00–67.00)	60.50 (56.75–72.25)	62.00 (54.00–66.00)	0.4467
Male sex (%)	26 (74.29)	18 (81.82)	8 (61.54)	0.1954
BMI (kg/m^2^) (IQR)	29.40 (26.30–32.30)	30.15 (26.53–33.03)	29.30 (24.25–30.80)	0.7779
Diabetes mellitus (%)	8 (22.86)	8 (36.36)	0 (0)	* 0.0124
Chronic renal failure (%)	5 (14.29)	2 (9.09)	3 (23.08)	0.2663
Smoker (%)	2 (5.71)	2 (9.09)	0 (0)	0.2762
Ex-smoker (%)	3 (8.57)	2 (9.09)	1 (7.69)	0.8905
LOS (days) (IQR)	38.00 (26.00–55.00)	38.50 (27.50–52.75)	30.00 (18.00–56.00)	0.5713
LOS ICU (days) (IQR)	19.00 (16.00–35.00)	23.50 (17.50–46.25)	17.00 (12.50–23.00)	* 0.0443
28-day mortality (%)	12 (34.29)	9 (40.91)	3 (23.08)	0.2967

Data are presented as *n* (%) or median (IQR). An unpaired *t*-test (two-tailed) was used for statistical analysis with * *p* < 0.05. IQR: interquartile ranges (Q1–Q3); BMI: body mass index; LOS: length of stay; ICU: intensive care unit.

**Table 2:** Patient characteristics of Cohort B.

	All (*n* = 80)	Increasing RNase 1 Levels (*n* = 48)	Decreasing RNase 1 Levels (*n* = 32)	*p*-Value
Age (year) (IQR)	64.00 (52.00–75.00)	63.00 (55.00–71.75)	68.00 (51.00–78.25)	0.5802
Male sex (%)	51 (63.75)	30 (62.5)	21 (65.63)	0.7791
BMI (kg/m^2^) (IQR)	28.49 (26.10–31.95)	27.73 (25.45–31.22)	29.40 (26.64–32.89)	0.5656
LOS (days) (IQR)	18.00 (12.25–34.00)	18.50 (12.25–38.50)	16.50 (10.75–27.00)	0.5515
LOS ICU (days) (IQR)	19.00 (12.00–38.75)	18.00 (12.00–35.00)	21.00 (12.50–44.50)	0.9218
ICU patients (%)	44 (55.00)	31 (64.58)	13 (40.63)	* 0.0351
28-day mortality (%)	22 (27.50)	18 (37.50)	4 (12.5)	* 0.0138

Data are presented as *n* (%) or median (IQR). An unpaired *t*-test (two-tailed) was used for statistical analysis with * *p* < 0.05. IQR: interquartile ranges (Q1–Q3); BMI: body mass index; LOS: length of stay; ICU: intensive care unit.

**Table 3:** Correlations of RNase 1 with various clinical parameters.

	**Increasing RNase 1 d2**		**Decreasing RNase 1 d2**	
**Variable**	**Pearson *r***	***p*-Value**	**Pearson *r***	***p*-Value**
**CRP**	0.0360	0.8909	−0.5112	0.1080
**PCT**	0.5570	* 0.0132	0.1131	0.7263
**IL-6**	−0.2412	0.3197	−0.2364	0.4839
**Leucocytes**	0.3793	0.0899	−0.3282	0.2977
**days of dialysis**	0.5038	* 0.0199	0.2462	0.4406
**Diuresis**	−0.6085	* 0.0044	−0.2475	0.4379
	**Increasing RNase 1 d4**		**Decreasing RNase 1 d4**	
**Variable**	**Pearson *r***	***p*-value**	**Pearson *r***	***p*-value**
**CRP**	−0.5576	* 0.0200	0.1304	0.7023
**PCT**	0.3239	0.1414	0.1752	0.5859
**IL-6**	0.1298	0.5855	0.3544	0.2849
**Leucocytes**	0.5044	* 0.0167	−0.0142	0.9650
**days of dialysis**	0.5812	* 0.0046	0.1999	0.5334
**Diuresis**	−0.6414	* 0.0013	−0.3839	0.2179

Shown are correlations of RNase 1 serum level of the increasing and decreasing groups at days 2 and 4 with various clinical parameters and correlation coefficient *r* with 95% confidence interval CI. *: significant correlations; CRP: C-reactive protein; IL-6: interleukin-6; PCT: procalcitonin.

## Data Availability

Not applicable.
